# 2,2-Dimethyl-2,3-dihydro-1*H*-perimidine

**DOI:** 10.1107/S1600536813000986

**Published:** 2013-01-19

**Authors:** Sarah Maloney, Alexandra M. Z. Slawin, J. Derek Woollins

**Affiliations:** a EaStCHEM School of Chemistry, University of St Andrews, St Andrews, Fife KY16 9ST, Scotland

## Abstract

The title compound, C_13_H_14_N_2_, was obtained from reaction of diaminona­phthalene with acetone. In both independent mol­ecules in the asymmetric unit, the tricyclic perimidine consists of a planar (r.m.s. deviations = 0.0125 and 0.0181 Å) naphthalene ring system and an envelope conformation C_4_N_2_ ringwith the NCN group hinged with respect to the naph­thalene backbone by 36.9 (2) and 41.3 (2)° in the two independent molecules. The methyl substituents are arranged approximately axial and equatorial on the apical C atom. In the crystal, one of the N—H groups of one independent mol­ecule is involved in classical N—H⋯N hydrogen bonding. Short inter­molecular (C/N)—H⋯π(arene) inter­actions, near the short T-shaped limit, link mol­ecules in the absence of strong acceptors.

## Related literature

For general background to perimidines and their biological activity, see: Shaabani & Maleki (2008 ▶); Sauer *et al.* (2006 ▶). For related structures, see: Martinez-Belmonte *et al.* (2010 ▶).
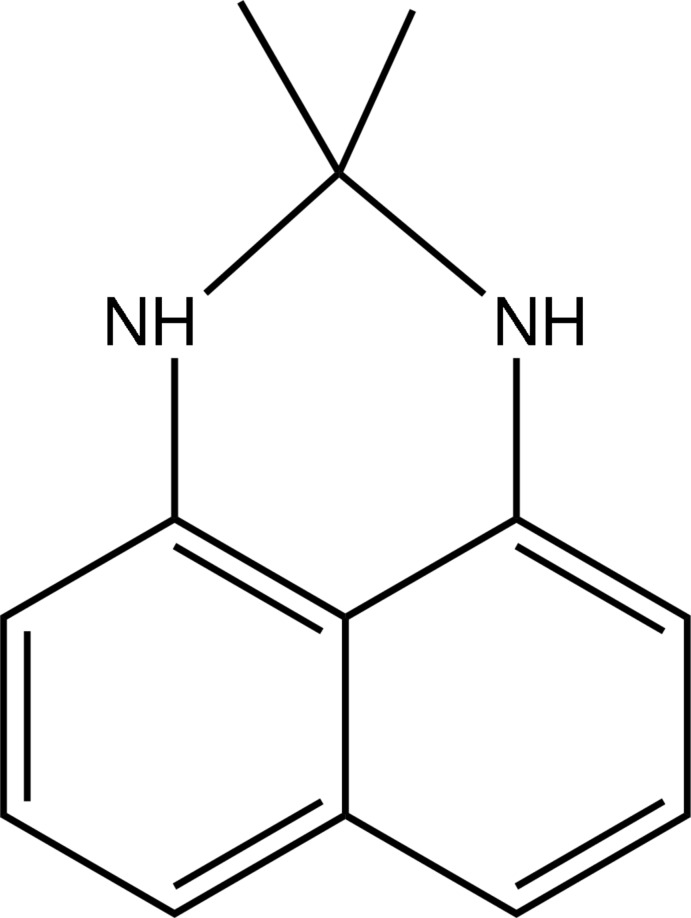


## Experimental

### 

#### Crystal data

C_13_H_14_N_2_
*M* *_r_* = 198.27
Monoclinic, 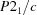

*a* = 16.261 (10) Å
*b* = 7.710 (4) Å
*c* = 17.483 (10) Å
β = 106.131 (12)°
*V* = 2105.6 (19) Å^3^
*Z* = 8
Mo *K*α radiation
μ = 0.08 mm^−1^
*T* = 93 K
0.12 × 0.12 × 0.12 mm

#### Data collection

Rigaku Mercury70 diffractometer
Absorption correction: multi-scan (*REQAB*; Rigaku, 1998 ▶) *T*_min_ = 0.706, *T*_max_ = 0.991
12841 measured reflections
3743 independent reflections
2356 reflections with *F* ^2^ > 2σ(*F* ^2^)
*R*_int_ = 0.072

#### Refinement

*R*[*F* ^2^ > 2σ(*F* ^2^)] = 0.056
*wR*(*F* ^2^) = 0.133
*S* = 0.96
3743 reflections
287 parameters
H atoms treated by a mixture of independent and constrained refinement
Δρ_max_ = 0.22 e Å^−3^
Δρ_min_ = −0.24 e Å^−3^

### 

Data collection: *CrystalClear-SM Expert* (Rigaku, 2009 ▶); cell refinement: *CrystalClear-SM Expert*; data reduction: *CrystalClear-SM Expert*; program(s) used to solve structure: *SIR2002* (Burla *et al.*, 2003 ▶); program(s) used to refine structure: *SHELXL97* (Sheldrick, 2008 ▶); molecular graphics: *CrystalStructure* (Rigaku, 2010 ▶); software used to prepare material for publication: *CrystalStructure*.

## Supplementary Material

Crystal structure: contains datablock(s) global, I. DOI: 10.1107/S1600536813000986/gg2107sup1.cif

Structure factors: contains datablock(s) I. DOI: 10.1107/S1600536813000986/gg2107Isup2.hkl

Supplementary material file. DOI: 10.1107/S1600536813000986/gg2107Isup3.cml

Additional supplementary materials:  crystallographic information; 3D view; checkCIF report

## Figures and Tables

**Table 1:** Hydrogen-bond geometry (Å, °) *Cg*1, *Cg*2 and *Cg*3 are the centroids of the C21–C25/C30, C25–C30 and C5–C10 rings, respectively.

*D*—H⋯*A*	*D*—H	H⋯*A*	*D*⋯*A*	*D*—H⋯*A*
N21—H21N⋯N29^i^	0.88 (2)	2.36 (2)	3.229 (4)	170 (2)
N1—H1N⋯*Cg*1^ii^	0.93 (3)	2.93 (3)	3.853 (3)	170 (2)
N9—H9N⋯*Cg*1	0.88 (4)	2.85 (2)	3.703 (3)	164 (2)
C12—H12*C*⋯*Cg*2	0.98	2.55	3.521 (3)	172
C26—H26⋯*Cg*3^iii^	0.95	2.53	3.456 (3)	164

Symmetry codes: (i) 
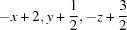
; (ii) 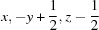; (iii) 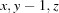.

**Fig. 1.**
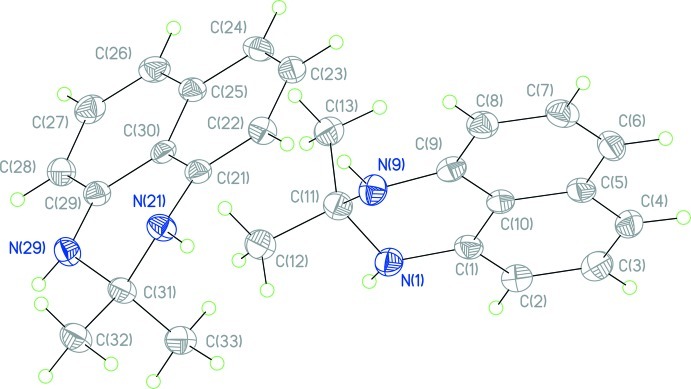
The structure of (1) with displacement ellipsoids drawn at the 50% probability level

## supplementary crystallographic information

### Comment

The title compound has a planar napthalene backbone with the the nitrogen atoms above this plane in both independent molecules (N1 0.133, N9 0.182, N21 0.155, N290.047 Å) and the carbon in the C4N2 ring being below this plane (C11 - 0.373, C31 - 0.488 Å). The six membered C_4_N_2_ ring is hinged about the N···N vector with the N1—C11—N9 and C21—C31—N29 planes being inclined by 36.9 and 41.0° respectively to the naphthalene planes.

### Experimental

A solution of 1,8-aminonapthalene (1.52 g, 0.01 mol s) and acetone (0.58 g, 0.78 ml, 0.01 mol s) was refluxed in ethanol (50 ml) for 2 h. The solution was then filtered, solvent evaporated to dryness under reduced pressure and recrystallized from boiling ethanol to give translucent crystals (mp *x^o^*C). (0.897 g, 0.0045 mol s, 45%). IR (KBr disc): 3295 ν(N—H), 1596 aromatic C—H, 1016, 646, 552, 477 cm^-1^. Raman (solid sample): 1490, 447, 160 cm^-1^. δ_H_ (270.0 MHz, CDCl_3_, Me_4_Si): 7.24–7.14 (4*H*, *m*, H_1_, H_2_), 6.47–6.44 (2*H*, *dd*, H_3_), 4.16 (2*H*, *brd s*, H_4_), 1.49 (6*H*, *s*, H_5_). δ_C_ (75.5 MHz, CDCl_3_, Me_4_Si): 140.4 (2 C, C_4_), 127.1 (2 C,*C*_3_), 117.2 (2 C, C_2_), 106.1 (2 C, C_1_), 64.7 (1 C, C_5_), 28.9 (2 C, C_6_). MS EI+: *m/z* 221.05 (*M*^+^ + Na)^+^ , 199.06 (*M*^+^ + H)^+^, 182.08 (*M*^+^ - Me)^+^. Found: C, 78.27; H, 6.93 N, 14.21. C_13_H_14_N_2_ requires C, 78.75; H, 7.12; N, 14.13%.

### Refinement

All H atoms were included in calculated positions (C—H distances are 0.98 Å for methyl H atoms, 0.95 Å for phenyl H atoms) and were refined as riding atoms with *U*_iso_(H) = 1.2 *U*_eq_(parent atom, methylene H atoms) or *U*_iso_(H) = 1.5 *U*_eq_(parent atom, methyl H atoms). The highest peak in the difference map is 0.94 Å from atom C29.

### Crystal data

**Table:**

C_13_H_14_N_2_	*F*(000) = 848.00
*M**_r_* = 198.27	*D*_x_ = 1.251 Mg m^−^^3^
Monoclinic, *P*2_1_/*c*	Mo *K*α radiation, λ = 0.71075 Å
Hall symbol: -P 2ybc	Cell parameters from 4713 reflections
*a* = 16.261 (10) Å	θ = 2.4–25.4°
*b* = 7.710 (4) Å	µ = 0.08 mm^−^^1^
*c* = 17.483 (10) Å	*T* = 93 K
β = 106.131 (12)°	Prism, colorless
*V* = 2105.6 (19) Å^3^	0.12 × 0.12 × 0.12 mm
*Z* = 8	

### Data collection

**Table:**

Rigaku Mercury70 diffractometer	2356 reflections with *F*^2^ > 2σ(*F*^2^)
Detector resolution: 14.629 pixels mm^-1^	*R*_int_ = 0.072
ω scans	θ_max_ = 25.1°
Absorption correction: multi-scan (*REQAB*; Rigaku, 1998)	*h* = −19→19
*T*_min_ = 0.706, *T*_max_ = 0.991	*k* = −9→9
12841 measured reflections	*l* = −20→16
3743 independent reflections	

### Refinement

**Table:**

Refinement on *F*^2^	Secondary atom site location: difference Fourier map
*R*[*F*^2^ > 2σ(*F*^2^)] = 0.056	Hydrogen site location: inferred from neighbouring sites
*wR*(*F*^2^) = 0.133	H atoms treated by a mixture of independent and constrained refinement
*S* = 0.96	*w* = 1/[σ^2^(*F*_o_^2^) + (0.0447*P*)^2^] where *P* = (*F*_o_^2^ + 2*F*_c_^2^)/3
3743 reflections	(Δ/σ)_max_ < 0.001
287 parameters	Δρ_max_ = 0.22 e Å^−^^3^
0 restraints	Δρ_min_ = −0.24 e Å^−^^3^
Primary atom site location: structure-invariant direct methods	

### Special details

**Table:**

Geometry. ENTER SPECIAL DETAILS OF THE MOLECULAR GEOMETRY
Refinement. Refinement was performed using all reflections. The weighted *R*-factor (*wR*) and goodness of fit (*S*) are based on *F*^2^. *R*-factor (gt) are based on *F*. The threshold expression of *F*^2^ > 2.0 σ(*F*^2^) is used only for calculating *R*-factor (gt).

### Fractional atomic coordinates and isotropic or equivalent isotropic displacement parameters (Å^2^)

**Table:**

	*x*	*y*	*z*	*U*_iso_*/*U*_eq_	
N1	0.74130 (13)	0.4938 (3)	0.32012 (12)	0.0251 (5)	
N9	0.72027 (13)	0.4474 (3)	0.44670 (12)	0.0253 (5)	
C1	0.67357 (15)	0.6139 (3)	0.29931 (14)	0.0239 (6)	
C2	0.65289 (15)	0.7024 (3)	0.22781 (14)	0.0257 (6)	
C3	0.58887 (16)	0.8304 (3)	0.21297 (15)	0.0293 (6)	
C4	0.54615 (16)	0.8709 (3)	0.26806 (15)	0.0276 (6)	
C5	0.56482 (15)	0.7808 (3)	0.34165 (14)	0.0245 (6)	
C6	0.52239 (16)	0.8170 (3)	0.40084 (15)	0.0296 (7)	
C7	0.54224 (15)	0.7241 (3)	0.47065 (15)	0.0304 (7)	
C8	0.60553 (16)	0.5958 (3)	0.48627 (15)	0.0280 (6)	
C9	0.64967 (15)	0.5602 (3)	0.43120 (14)	0.0240 (6)	
C10	0.62879 (15)	0.6508 (3)	0.35711 (14)	0.0225 (6)	
C11	0.73892 (16)	0.3581 (3)	0.37924 (14)	0.0255 (6)	
C12	0.82772 (15)	0.2789 (3)	0.40713 (15)	0.0301 (7)	
C13	0.67145 (15)	0.2193 (3)	0.34472 (14)	0.0286 (7)	
H2	0.6818	0.6768	0.1888	0.0309*	
H3	0.5749	0.8904	0.1636	0.0351*	
H4	0.5039	0.9595	0.2569	0.0331*	
H6	0.4802	0.9056	0.3921	0.0355*	
H7	0.5123	0.7475	0.5091	0.0364*	
H8	0.6181	0.5331	0.5349	0.0336*	
H12A	0.8692	0.3688	0.4320	0.0361*	
H12B	0.8434	0.2296	0.3614	0.0361*	
H12C	0.8280	0.1872	0.4460	0.0361*	
H13A	0.6846	0.1634	0.2992	0.0343*	
H13B	0.6148	0.2735	0.3272	0.0343*	
H13C	0.6718	0.1322	0.3856	0.0343*	
H9n	0.7242 (16)	0.381 (3)	0.4888 (15)	0.035 (8)*	
H1n	0.7596 (16)	0.455 (3)	0.2770 (16)	0.043 (8)*	
N21	0.95216 (13)	0.2662 (3)	0.72018 (12)	0.0237 (5)	
N29	1.01370 (14)	0.0717 (3)	0.64843 (12)	0.0240 (5)	
C21	0.86940 (15)	0.2322 (3)	0.67450 (13)	0.0213 (6)	
C22	0.79708 (15)	0.3089 (3)	0.68648 (14)	0.0236 (6)	
C23	0.71473 (16)	0.2555 (3)	0.64207 (14)	0.0255 (6)	
C24	0.70369 (15)	0.1272 (3)	0.58569 (14)	0.0259 (6)	
C25	0.77628 (15)	0.0457 (3)	0.57089 (14)	0.0222 (6)	
C26	0.76848 (16)	−0.0902 (3)	0.51485 (14)	0.0267 (6)	
C27	0.84036 (16)	−0.1676 (3)	0.50372 (14)	0.0277 (6)	
C28	0.92278 (16)	−0.1122 (3)	0.54540 (14)	0.0252 (6)	
C29	0.93299 (15)	0.0203 (3)	0.60052 (14)	0.0225 (6)	
C30	0.85910 (15)	0.1007 (3)	0.61425 (13)	0.0200 (6)	
C31	1.02152 (15)	0.2496 (3)	0.68135 (14)	0.0235 (6)	
C32	1.10655 (15)	0.2637 (3)	0.74320 (15)	0.0303 (7)	
C33	1.01217 (16)	0.3872 (3)	0.61631 (14)	0.0281 (7)	
H21n	0.9598 (16)	0.359 (3)	0.7505 (15)	0.033 (8)*	
H29n	1.0552 (17)	0.048 (3)	0.6295 (15)	0.039 (9)*	
H22	0.8030	0.3983	0.7250	0.0283*	
H23	0.6659	0.3094	0.6514	0.0305*	
H24	0.6476	0.0928	0.5566	0.0311*	
H26	0.7133	−0.1279	0.4848	0.0320*	
H27	0.8341	−0.2608	0.4670	0.0332*	
H28	0.9716	−0.1657	0.5357	0.0303*	
H32A	1.1527	0.2410	0.7185	0.0364*	
H32B	1.1093	0.1785	0.7854	0.0364*	
H32C	1.1131	0.3807	0.7660	0.0364*	
H33A	1.0152	0.5030	0.6401	0.0337*	
H33B	0.9568	0.3729	0.5763	0.0337*	
H33C	1.0584	0.3738	0.5909	0.0337*	

### Atomic displacement parameters (Å^2^)

**Table:**

	*U*^11^	*U*^22^	*U*^33^	*U*^12^	*U*^13^	*U*^23^
N1	0.0277 (14)	0.0244 (11)	0.0255 (13)	0.0014 (10)	0.0114 (11)	0.0024 (10)
N9	0.0296 (14)	0.0263 (12)	0.0200 (13)	0.0024 (10)	0.0065 (11)	0.0030 (10)
C1	0.0197 (15)	0.0222 (13)	0.0300 (15)	−0.0044 (11)	0.0073 (12)	−0.0033 (11)
C2	0.0266 (16)	0.0271 (13)	0.0236 (14)	−0.0037 (12)	0.0072 (13)	−0.0006 (11)
C3	0.0304 (16)	0.0283 (14)	0.0254 (15)	−0.0047 (12)	0.0016 (13)	0.0028 (11)
C4	0.0243 (15)	0.0222 (13)	0.0338 (16)	−0.0018 (11)	0.0038 (13)	0.0008 (12)
C5	0.0197 (14)	0.0227 (13)	0.0301 (15)	−0.0053 (11)	0.0049 (12)	−0.0033 (11)
C6	0.0227 (15)	0.0279 (14)	0.0383 (17)	0.0020 (12)	0.0087 (14)	−0.0029 (12)
C7	0.0278 (16)	0.0334 (15)	0.0337 (16)	−0.0032 (12)	0.0149 (14)	−0.0030 (12)
C8	0.0292 (16)	0.0295 (14)	0.0283 (16)	−0.0024 (12)	0.0130 (13)	0.0012 (11)
C9	0.0257 (16)	0.0211 (13)	0.0245 (15)	−0.0055 (11)	0.0063 (13)	−0.0012 (10)
C10	0.0202 (15)	0.0199 (12)	0.0264 (14)	−0.0047 (11)	0.0050 (12)	−0.0019 (11)
C11	0.0246 (15)	0.0269 (13)	0.0246 (15)	−0.0007 (11)	0.0062 (13)	−0.0006 (11)
C12	0.0272 (16)	0.0318 (14)	0.0316 (16)	−0.0002 (12)	0.0088 (13)	0.0015 (12)
C13	0.0280 (16)	0.0259 (13)	0.0313 (16)	−0.0016 (12)	0.0071 (13)	0.0013 (11)
N21	0.0212 (13)	0.0263 (12)	0.0237 (13)	−0.0023 (10)	0.0066 (11)	−0.0048 (9)
N29	0.0204 (13)	0.0239 (11)	0.0271 (13)	0.0013 (10)	0.0054 (11)	−0.0041 (9)
C21	0.0203 (15)	0.0220 (13)	0.0213 (14)	−0.0019 (11)	0.0055 (12)	0.0038 (10)
C22	0.0233 (15)	0.0251 (13)	0.0227 (14)	−0.0007 (11)	0.0069 (12)	−0.0021 (11)
C23	0.0249 (16)	0.0282 (14)	0.0260 (15)	0.0025 (11)	0.0115 (13)	0.0073 (11)
C24	0.0190 (15)	0.0329 (14)	0.0261 (15)	−0.0030 (11)	0.0068 (13)	0.0047 (12)
C25	0.0204 (15)	0.0250 (13)	0.0219 (14)	−0.0015 (11)	0.0071 (12)	0.0049 (11)
C26	0.0244 (15)	0.0253 (13)	0.0285 (16)	−0.0070 (11)	0.0043 (13)	−0.0015 (11)
C27	0.0318 (17)	0.0213 (13)	0.0303 (16)	−0.0042 (12)	0.0092 (13)	−0.0050 (11)
C28	0.0256 (16)	0.0239 (13)	0.0274 (15)	−0.0005 (11)	0.0093 (13)	−0.0021 (11)
C29	0.0213 (15)	0.0234 (13)	0.0233 (14)	−0.0014 (11)	0.0069 (12)	0.0034 (11)
C30	0.0229 (15)	0.0168 (12)	0.0212 (14)	−0.0017 (11)	0.0078 (12)	0.0034 (10)
C31	0.0197 (15)	0.0251 (13)	0.0264 (14)	−0.0018 (11)	0.0074 (12)	−0.0033 (11)
C32	0.0227 (15)	0.0332 (14)	0.0351 (16)	−0.0022 (12)	0.0081 (13)	−0.0054 (12)
C33	0.0278 (16)	0.0281 (14)	0.0297 (16)	−0.0033 (12)	0.0102 (13)	−0.0024 (11)

### Geometric parameters (Å, º)

**Table:**

N1—C1	1.407 (3)	C29—C30	1.430 (4)
N1—C11	1.479 (4)	C31—C32	1.504 (3)
N9—C9	1.406 (3)	C31—C33	1.531 (4)
N9—C11	1.468 (4)	N1—H1n	0.93 (3)
C1—C2	1.381 (4)	N9—H9n	0.88 (3)
C1—C10	1.429 (4)	C2—H2	0.950
C2—C3	1.405 (4)	C3—H3	0.950
C3—C4	1.371 (5)	C4—H4	0.950
C4—C5	1.418 (4)	C6—H6	0.950
C5—C6	1.422 (4)	C7—H7	0.950
C5—C10	1.416 (4)	C8—H8	0.950
C6—C7	1.374 (4)	C12—H12A	0.980
C7—C8	1.398 (4)	C12—H12B	0.980
C8—C9	1.379 (4)	C12—H12C	0.980
C9—C10	1.427 (4)	C13—H13A	0.980
C11—C12	1.518 (4)	C13—H13B	0.980
C11—C13	1.532 (4)	C13—H13C	0.980
N21—C21	1.386 (3)	N21—H21n	0.88 (3)
N21—C31	1.475 (4)	N29—H29n	0.85 (3)
N29—C29	1.404 (3)	C22—H22	0.950
N29—C31	1.480 (3)	C23—H23	0.950
C21—C22	1.383 (4)	C24—H24	0.950
C21—C30	1.438 (3)	C26—H26	0.950
C22—C23	1.410 (4)	C27—H27	0.950
C23—C24	1.373 (4)	C28—H28	0.950
C24—C25	1.423 (4)	C32—H32A	0.980
C25—C26	1.416 (4)	C32—H32B	0.980
C25—C30	1.414 (3)	C32—H32C	0.980
C26—C27	1.374 (4)	C33—H33A	0.980
C27—C28	1.402 (4)	C33—H33B	0.980
C28—C29	1.382 (4)	C33—H33C	0.980
			
N1···C9	2.804 (4)	H8···C7^xi^	3.2563
N9···C1	2.789 (4)	H8···H2^xii^	3.4300
C1···C4	2.808 (4)	H8···H3^xii^	2.6041
C1···C13	3.147 (4)	H8···H7^xi^	2.9778
C2···C5	2.822 (4)	H8···C23	2.9872
C3···C10	2.789 (4)	H8···C24	3.4399
C5···C8	2.817 (4)	H8···H23	2.6159
C6···C9	2.806 (4)	H8···H24	3.4350
C7···C10	2.791 (4)	H8···H26^iii^	3.2782
C9···C13	3.101 (4)	H12A···C33	3.4131
C10···C11	2.840 (4)	H12A···C33^ii^	2.9801
C10···C13	3.418 (4)	H12A···H27^iii^	3.0090
N21···C29	2.776 (4)	H12A···H28^xiii^	2.9433
N29···C21	2.800 (4)	H12A···H33A^ii^	2.7227
C21···C24	2.829 (4)	H12A···H33B	2.5307
C21···C33	3.028 (4)	H12A···H33B^ii^	3.4961
C22···C25	2.817 (4)	H12A···H33C	3.5273
C23···C30	2.794 (4)	H12A···H33C^ii^	2.3971
C25···C28	2.819 (4)	H12B···N21^i^	3.4143
C26···C29	2.810 (4)	H12B···N29^xiii^	3.3243
C27···C30	2.789 (4)	H12B···C21^i^	3.4228
C29···C33	3.089 (4)	H12B···C22^i^	2.9547
C30···C31	2.819 (4)	H12B···H21n^i^	3.1389
C30···C33	3.321 (4)	H12B···H29n^xiii^	2.6812
N1···C22^i^	3.593 (4)	H12B···H22^i^	2.4947
N1···C23^i^	3.581 (4)	H12B···H28^xiii^	3.0861
N1···C32^ii^	3.516 (4)	H12B···H33A^ii^	3.0943
N9···C24	3.526 (4)	H12B···H33C^ii^	3.4433
N9···C27^iii^	3.541 (4)	H12C···C25	2.7718
C4···C13^iii^	3.413 (4)	H12C···C26	2.7575
C8···C26^iii^	3.521 (4)	H12C···C27	2.9033
C9···C26^iii^	3.401 (4)	H12C···C28	3.0393
C13···C4^iv^	3.413 (4)	H12C···C29	3.0507
N21···N29^v^	3.229 (3)	H12C···C30	2.9201
N29···N21^vi^	3.229 (3)	H12C···H29n^xiii^	3.1687
C22···N1^vii^	3.593 (4)	H12C···H26	3.2466
C23···N1^vii^	3.581 (4)	H12C···H27	3.4716
C24···N9	3.526 (4)	H12C···H28^xiii^	3.1900
C26···C8^iv^	3.521 (4)	H12C···H33B	2.9969
C26···C9^iv^	3.401 (4)	H13A···C3^iv^	3.1595
C27···N9^iv^	3.541 (4)	H13A···C4^iv^	3.1260
C29···C32^vi^	3.573 (4)	H13A···H3^iv^	3.2995
C30···C32^vi^	3.535 (4)	H13A···H4^iv^	3.2321
C32···N1^ii^	3.516 (4)	H13A···C22^i^	3.0453
C32···C29^v^	3.573 (4)	H13A···C23^i^	2.9871
C32···C30^v^	3.535 (4)	H13A···H22^i^	2.6455
N1···H2	2.6386	H13A···H23^i^	2.5254
N1···H12A	2.6111	H13B···C3^viii^	3.2186
N1···H12B	2.6002	H13B···C4^iv^	3.3632
N1···H12C	3.2706	H13B···C4^viii^	2.7866
N1···H13A	2.6998	H13B···C5^viii^	3.5298
N1···H13B	2.6978	H13B···H3^viii^	3.2593
N1···H13C	3.3294	H13B···H4^iv^	3.0647
N1···H9n	3.16 (3)	H13B···H4^viii^	2.5235
N9···H8	2.6451	H13B···H23^i^	3.4602
N9···H12A	2.5773	H13C···C4^iv^	3.1820
N9···H12B	3.2719	H13C···C5^iv^	3.1957
N9···H12C	2.6654	H13C···C6^iv^	3.4981
N9···H13A	3.3094	H13C···H4^iv^	3.2978
N9···H13B	2.6666	H13C···C24	3.3904
N9···H13C	2.6832	H13C···C25	3.2808
N9···H1n	3.20 (3)	H13C···C26	2.9227
C1···H3	3.2611	H13C···H24	3.1356
C1···H13A	3.4788	H13C···H26	2.6146
C1···H13B	2.8816	H9n···C22	3.37 (3)
C2···H4	3.2775	H9n···C23	2.89 (3)
C2···H1n	2.56 (3)	H9n···C24	2.67 (3)
C4···H2	3.2743	H9n···C25	2.96 (3)
C4···H6	2.6865	H9n···C30	3.41 (3)
C5···H3	3.2735	H9n···H23	3.2829
C5···H7	3.2793	H9n···H24	2.9522
C6···H4	2.6848	H9n···H27^iii^	3.3681
C6···H8	3.2684	H1n···C21^i^	3.20 (3)
C8···H6	3.2718	H1n···C22^i^	2.75 (3)
C8···H9n	2.53 (3)	H1n···C23^i^	2.79 (3)
C9···H7	3.2583	H1n···C24^i^	3.27 (3)
C9···H13B	2.8178	H1n···C32^ii^	3.16 (3)
C9···H13C	3.4372	H1n···H22^i^	3.0137
C10···H2	3.2938	H1n···H23^i^	3.0707
C10···H4	3.2984	H1n···H32A^ii^	2.7368
C10···H6	3.3005	H1n···H32C^ii^	2.7066
C10···H8	3.2887	H1n···H33A^ii^	3.5589
C10···H13B	2.9537	H1n···H33C^ii^	3.4702
C10···H9n	3.17 (3)	N21···H12B^vii^	3.4143
C10···H1n	3.23 (3)	N21···H21n^vi^	3.43 (3)
C12···H13A	2.7073	N21···H29n^v^	3.44 (3)
C12···H13B	3.3548	N21···H32B^v^	3.3261
C12···H13C	2.7058	N21···H32C^vi^	3.1878
C12···H9n	2.61 (3)	N21···H33A^vi^	3.1063
C12···H1n	2.61 (3)	N29···H12B^xiii^	3.3243
C13···H12A	3.3566	N29···H21n^vi^	2.36 (3)
C13···H12B	2.7297	N29···H22^vi^	3.4504
C13···H12C	2.6775	N29···H28^xiii^	3.3733
C13···H9n	2.73 (3)	N29···H32C^vi^	3.2210
C13···H1n	2.77 (3)	C21···H12B^vii^	3.4228
H2···H3	2.3459	C21···H1n^vii^	3.20 (3)
H2···H1n	2.4150	C21···H32B^v^	3.5102
H3···H4	2.3076	C21···H32C^vi^	2.8885
H4···H6	2.5325	C22···H12B^vii^	2.9547
H6···H7	2.3131	C22···H13A^vii^	3.0453
H7···H8	2.3375	C22···H9n	3.37 (3)
H8···H9n	2.4025	C22···H1n^vii^	2.75 (3)
H12A···H13C	3.5835	C22···H32B^v^	3.2046
H12A···H9n	2.8024	C23···H2^vii^	3.5084
H12A···H1n	2.8756	C23···H6^xi^	3.3009
H12B···H13A	2.5585	C23···H8	2.9872
H12B···H13C	3.0303	C23···H13A^vii^	2.9871
H12B···H9n	3.5319	C23···H9n	2.89 (3)
H12B···H1n	2.4339	C23···H1n^vii^	2.79 (3)
H12C···H13A	2.9546	C24···H2^vii^	3.0379
H12C···H13B	3.5735	C24···H6^xi^	3.1308
H12C···H13C	2.5009	C24···H7^xi^	3.5757
H12C···H9n	2.5185	C24···H8	3.4399
H12C···H1n	3.5197	C24···H13C	3.3904
H13A···H1n	2.6343	C24···H9n	2.67 (3)
H13B···H9n	3.0071	C24···H1n^vii^	3.27 (3)
H13B···H1n	3.0686	C25···H2^vii^	3.3639
H13C···H9n	2.6096	C25···H12C	2.7718
N21···H29n	3.10 (3)	C25···H13C	3.2808
N21···H22	2.6543	C25···H9n	2.96 (3)
N21···H32A	3.2749	C25···H32C^vi^	3.1837
N21···H32B	2.5816	C26···H12C	2.7575
N21···H32C	2.6660	C26···H13C	2.9227
N21···H33A	2.6732	C27···H12C	2.9033
N21···H33B	2.6669	C27···H29n^xiii^	3.37 (3)
N21···H33C	3.3100	C27···H33C^xiii^	3.0795
N29···H21n	3.12 (3)	C28···H12C	3.0393
N29···H28	2.6364	C28···H21n^vi^	3.56 (3)
N29···H32A	2.6025	C28···H29n^xiii^	3.21 (3)
N29···H32B	2.6007	C28···H28^xiii^	3.3031
N29···H32C	3.2663	C28···H32B^vi^	3.5320
N29···H33A	3.3292	C28···H32C^vi^	3.5052
N29···H33B	2.6812	C28···H33A^iv^	3.5240
N29···H33C	2.7158	C28···H33C^xiii^	3.2005
C21···H23	3.2755	C29···H12C	3.0507
C21···H33A	3.3374	C29···H21n^vi^	2.97 (3)
C21···H33B	2.7385	C29···H28^xiii^	3.3757
C22···H21n	2.60 (3)	C29···H32B^vi^	3.4857
C22···H24	3.2822	C29···H32C^vi^	2.8530
C24···H22	3.2747	C30···H12C	2.9201
C24···H26	2.6750	C30···H9n	3.41 (3)
C25···H23	3.2772	C30···H32C^vi^	2.6341
C25···H27	3.2739	C31···H21n^vi^	3.22 (3)
C26···H24	2.6798	C32···H2^ii^	3.3457
C26···H28	3.2725	C32···H1n^ii^	3.16 (3)
C28···H29n	2.56 (3)	C32···H21n^vi^	3.31 (3)
C28···H26	3.2757	C32···H22^vi^	3.1551
C29···H27	3.2628	C33···H12A	3.4131
C29···H33B	2.7948	C33···H12A^ii^	2.9801
C29···H33C	3.4368	C33···H27^xiii^	3.3678
C30···H21n	3.19 (3)	C33···H28^xiii^	3.2317
C30···H29n	3.15 (3)	H21n···H12B^vii^	3.1389
C30···H22	3.2913	H21n···N21^v^	3.43 (3)
C30···H24	3.3044	H21n···N29^v^	2.36 (3)
C30···H26	3.2958	H21n···C28^v^	3.56 (3)
C30···H28	3.2927	H21n···C29^v^	2.97 (3)
C30···H33B	2.8207	H21n···C31^v^	3.22 (3)
C32···H21n	2.54 (3)	H21n···C32^v^	3.31 (3)
C32···H29n	2.55 (3)	H21n···H29n^v^	2.62 (4)
C32···H33A	2.7154	H21n···H28^v^	3.5955
C32···H33B	3.3464	H21n···H32A^v^	3.5842
C32···H33C	2.6955	H21n···H32B^v^	2.7055
C33···H21n	2.72 (3)	H21n···H33A^vi^	3.3080
C33···H29n	2.70 (3)	H29n···C12^xiii^	3.33 (3)
C33···H32A	2.7227	H29n···H12B^xiii^	2.6812
C33···H32B	3.3512	H29n···H12C^xiii^	3.1687
C33···H32C	2.6755	H29n···N21^vi^	3.44 (3)
H21n···H22	2.4816	H29n···C27^xiii^	3.37 (3)
H21n···H32A	3.4591	H29n···C28^xiii^	3.21 (3)
H21n···H32B	2.7206	H29n···H21n^vi^	2.62 (4)
H21n···H32C	2.4382	H29n···H22^vi^	3.1411
H21n···H33A	2.5955	H29n···H27^xiii^	3.2349
H21n···H33B	3.0340	H29n···H28^xiii^	2.9429
H21n···H33C	3.5867	H22···C12^vii^	3.3848
H29n···H28	2.4525	H22···C13^vii^	3.5020
H29n···H32A	2.4030	H22···H12B^vii^	2.4947
H29n···H32B	2.8080	H22···H13A^vii^	2.6455
H29n···H32C	3.4507	H22···H1n^vii^	3.0137
H29n···H33A	3.5809	H22···N29^v^	3.4504
H29n···H33B	2.9771	H22···C32^v^	3.1551
H29n···H33C	2.6048	H22···H29n^v^	3.1411
H22···H23	2.3458	H22···H32A^v^	2.8427
H23···H24	2.3130	H22···H32B^v^	2.6236
H24···H26	2.5211	H23···C3^xii^	3.3451
H26···H27	2.3115	H23···C6^xi^	3.0985
H27···H28	2.3423	H23···C7^xi^	3.4722
H32A···H33A	3.0419	H23···C8	3.5491
H32A···H33B	3.5989	H23···C13^vii^	3.3636
H32A···H33C	2.5440	H23···H3^xii^	2.7873
H32B···H33A	3.5913	H23···H6^xi^	2.8223
H32B···H33C	3.5996	H23···H7^xi^	3.4557
H32C···H33A	2.5146	H23···H8	2.6159
H32C···H33B	3.5739	H23···H13A^vii^	2.5254
H32C···H33C	2.9414	H23···H13B^vii^	3.4602
N1···H27^iii^	3.2088	H23···H9n	3.2829
N1···H32A^ii^	2.8729	H23···H1n^vii^	3.0707
N1···H32C^ii^	3.2841	H24···C6^xi^	3.1342
N1···H33C^ii^	3.3541	H24···C7^iv^	3.4416
N9···H26^iii^	3.3501	H24···C7^xi^	3.3068
N9···H27^iii^	2.8726	H24···H2^vii^	3.0432
C1···H4^viii^	3.0226	H24···H6^xi^	2.4821
C1···H27^iii^	3.4768	H24···H7^iv^	3.4063
C1···H32A^ii^	3.1334	H24···H7^xi^	2.8182
C2···H4^viii^	3.2340	H24···H8	3.4350
C2···H6^viii^	3.4343	H24···H13C	3.1356
C2···H32A^ii^	3.0689	H24···H9n	2.9522
C3···H4^viii^	3.3428	H26···N9^iv^	3.3501
C3···H7^ix^	3.4852	H26···C5^iv^	3.0389
C3···H8^ix^	3.4420	H26···C6^iv^	3.0731
C3···H13A^iii^	3.1595	H26···C7^iv^	2.9531
C3···H13B^x^	3.2186	H26···C8^iv^	2.7632
C3···H23^ix^	3.3451	H26···C9^iv^	2.6822
C4···H4^viii^	3.2736	H26···C10^iv^	2.8428
C4···H13A^iii^	3.1260	H26···C13	3.5643
C4···H13B^iii^	3.3632	H26···H7^iv^	3.5435
C4···H13B^x^	2.7866	H26···H8^iv^	3.2782
C4···H13C^iii^	3.1820	H26···H12C	3.2466
C5···H4^viii^	3.0475	H26···H13C	2.6146
C5···H13B^x^	3.5298	H27···N1^iv^	3.2088
C5···H13C^iii^	3.1957	H27···N9^iv^	2.8726
C5···H26^iii^	3.0389	H27···C1^iv^	3.4768
C6···H13C^iii^	3.4981	H27···C9^iv^	3.2013
C6···H23^xi^	3.0985	H27···C10^iv^	3.4259
C6···H24^xi^	3.1342	H27···C11^iv^	3.4733
C6···H26^iii^	3.0731	H27···H12A^iv^	3.0090
C7···H3^xii^	3.3823	H27···H12C	3.4716
C7···H8^xi^	3.2563	H27···H9n^iv^	3.3681
C7···H23^xi^	3.4722	H27···C33^xiii^	3.3678
C7···H24^iii^	3.4416	H27···H29n^xiii^	3.2349
C7···H24^xi^	3.3068	H27···H32A^xiii^	3.3116
C7···H26^iii^	2.9531	H27···H33C^xiii^	2.4122
C8···H3^xii^	3.2750	H28···C12^xiii^	3.2555
C8···H7^xi^	3.2821	H28···H12A^xiii^	2.9433
C8···H23	3.5491	H28···H12B^xiii^	3.0861
C8···H26^iii^	2.7632	H28···H12C^xiii^	3.1900
C9···H26^iii^	2.6822	H28···N29^xiii^	3.3733
C9···H27^iii^	3.2013	H28···C28^xiii^	3.3031
C10···H4^viii^	2.9010	H28···C29^xiii^	3.3757
C10···H26^iii^	2.8428	H28···C33^xiii^	3.2317
C10···H27^iii^	3.4259	H28···H21n^vi^	3.5955
C11···H27^iii^	3.4733	H28···H29n^xiii^	2.9429
C12···H29n^xiii^	3.33 (3)	H28···H28^xiii^	3.0967
C12···H22^i^	3.3848	H28···H33A^iv^	3.1040
C12···H28^xiii^	3.2555	H28···H33B^xiii^	3.0022
C12···H33A^ii^	3.3470	H28···H33C^xiii^	2.6672
C12···H33B	3.1989	H32A···N1^ii^	2.8729
C12···H33C^ii^	3.2500	H32A···C1^ii^	3.1334
C13···H4^iv^	3.3865	H32A···C2^ii^	3.0689
C13···H4^viii^	3.4499	H32A···H2^ii^	2.8014
C13···H22^i^	3.5020	H32A···H1n^ii^	2.7368
C13···H23^i^	3.3636	H32A···H21n^vi^	3.5842
C13···H26	3.5643	H32A···H22^vi^	2.8427
H2···H6^viii^	3.3533	H32A···H27^xiii^	3.3116
H2···H8^ix^	3.4300	H32B···H2^ii^	3.4840
H2···C23^i^	3.5084	H32B···N21^vi^	3.3261
H2···C24^i^	3.0379	H32B···C21^vi^	3.5102
H2···C25^i^	3.3639	H32B···C22^vi^	3.2046
H2···C32^ii^	3.3457	H32B···C28^v^	3.5320
H2···H24^i^	3.0432	H32B···C29^v^	3.4857
H2···H32A^ii^	2.8014	H32B···H21n^vi^	2.7055
H2···H32B^ii^	3.4840	H32B···H22^vi^	2.6236
H2···H32C^ii^	3.2366	H32B···H33A^vi^	3.0150
H3···C7^ix^	3.3823	H32C···N1^ii^	3.2841
H3···C8^ix^	3.2750	H32C···H2^ii^	3.2366
H3···H7^ix^	2.8157	H32C···H1n^ii^	2.7066
H3···H8^ix^	2.6041	H32C···N21^v^	3.1878
H3···H13A^iii^	3.2995	H32C···N29^v^	3.2210
H3···H13B^x^	3.2593	H32C···C21^v^	2.8885
H3···H23^ix^	2.7873	H32C···C25^v^	3.1837
H4···C1^x^	3.0226	H32C···C28^v^	3.5052
H4···C2^x^	3.2340	H32C···C29^v^	2.8530
H4···C3^x^	3.3428	H32C···C30^v^	2.6341
H4···C4^x^	3.2736	H33A···C12^ii^	3.3470
H4···C5^x^	3.0475	H33A···H12A^ii^	2.7227
H4···C10^x^	2.9010	H33A···H12B^ii^	3.0943
H4···C13^iii^	3.3865	H33A···H1n^ii^	3.5589
H4···C13^x^	3.4499	H33A···N21^v^	3.1063
H4···H13A^iii^	3.2321	H33A···C28^iii^	3.5240
H4···H13B^iii^	3.0647	H33A···H21n^v^	3.3080
H4···H13B^x^	2.5235	H33A···H28^iii^	3.1040
H4···H13C^iii^	3.2978	H33A···H32B^v^	3.0150
H6···C2^x^	3.4343	H33B···C12	3.1989
H6···H2^x^	3.3533	H33B···H12A	2.5307
H6···H7^xiv^	3.1679	H33B···H12A^ii^	3.4961
H6···C23^xi^	3.3009	H33B···H12C	2.9969
H6···C24^xi^	3.1308	H33B···H28^xiii^	3.0022
H6···H23^xi^	2.8223	H33B···H33C^ii^	3.4670
H6···H24^xi^	2.4821	H33C···N1^ii^	3.3541
H7···C3^xii^	3.4852	H33C···C12^ii^	3.2500
H7···C8^xi^	3.2821	H33C···H12A	3.5273
H7···H3^xii^	2.8157	H33C···H12A^ii^	2.3971
H7···H6^xiv^	3.1679	H33C···H12B^ii^	3.4433
H7···H8^xi^	2.9778	H33C···H1n^ii^	3.4702
H7···C24^xi^	3.5757	H33C···C27^xiii^	3.0795
H7···H23^xi^	3.4557	H33C···C28^xiii^	3.2005
H7···H24^iii^	3.4063	H33C···H27^xiii^	2.4122
H7···H24^xi^	2.8182	H33C···H28^xiii^	2.6672
H7···H26^iii^	3.5435	H33C···H33B^ii^	3.4670
H8···C3^xii^	3.4420		
			
C1—N1—C11	118.4 (3)	C9—N9—H9n	112.7 (18)
C9—N9—C11	118.37 (19)	C11—N9—H9n	114.5 (17)
N1—C1—C2	122.4 (3)	C1—C2—H2	120.147
N1—C1—C10	117.7 (2)	C3—C2—H2	120.144
C2—C1—C10	119.9 (2)	C2—C3—H3	119.198
C1—C2—C3	119.7 (3)	C4—C3—H3	119.197
C2—C3—C4	121.6 (3)	C3—C4—H4	119.883
C3—C4—C5	120.2 (3)	C5—C4—H4	119.895
C4—C5—C6	122.6 (3)	C5—C6—H6	119.925
C4—C5—C10	118.7 (3)	C7—C6—H6	119.912
C6—C5—C10	118.6 (3)	C6—C7—H7	119.315
C5—C6—C7	120.2 (3)	C8—C7—H7	119.307
C6—C7—C8	121.4 (3)	C7—C8—H8	119.937
C7—C8—C9	120.1 (3)	C9—C8—H8	119.924
N9—C9—C8	123.0 (2)	C11—C12—H12A	109.477
N9—C9—C10	117.0 (3)	C11—C12—H12B	109.468
C8—C9—C10	119.8 (2)	C11—C12—H12C	109.472
C1—C10—C5	119.9 (2)	H12A—C12—H12B	109.474
C1—C10—C9	120.3 (2)	H12A—C12—H12C	109.472
C5—C10—C9	119.8 (3)	H12B—C12—H12C	109.463
N1—C11—N9	106.33 (18)	C11—C13—H13A	109.472
N1—C11—C12	107.6 (3)	C11—C13—H13B	109.473
N1—C11—C13	111.97 (18)	C11—C13—H13C	109.473
N9—C11—C12	108.72 (19)	H13A—C13—H13B	109.459
N9—C11—C13	111.0 (3)	H13A—C13—H13C	109.477
C12—C11—C13	111.01 (19)	H13B—C13—H13C	109.473
C21—N21—C31	117.6 (2)	C21—N21—H21n	116.9 (16)
C29—N29—C31	117.32 (19)	C31—N21—H21n	110.5 (18)
N21—C21—C22	124.2 (2)	C29—N29—H29n	114.8 (16)
N21—C21—C30	116.9 (3)	C31—N29—H29n	111.0 (16)
C22—C21—C30	118.8 (2)	C21—C22—H22	119.693
C21—C22—C23	120.6 (3)	C23—C22—H22	119.705
C22—C23—C24	121.4 (3)	C22—C23—H23	119.309
C23—C24—C25	119.9 (2)	C24—C23—H23	119.313
C24—C25—C26	122.2 (2)	C23—C24—H24	120.020
C24—C25—C30	119.0 (2)	C25—C24—H24	120.040
C26—C25—C30	118.8 (3)	C25—C26—H26	119.885
C25—C26—C27	120.2 (2)	C27—C26—H26	119.879
C26—C27—C28	121.5 (3)	C26—C27—H27	119.268
C27—C28—C29	120.0 (3)	C28—C27—H27	119.271
N29—C29—C28	122.4 (3)	C27—C28—H28	120.016
N29—C29—C30	117.9 (2)	C29—C28—H28	120.012
C28—C29—C30	119.6 (2)	C31—C32—H32A	109.469
C21—C30—C25	120.2 (3)	C31—C32—H32B	109.478
C21—C30—C29	119.7 (2)	C31—C32—H32C	109.473
C25—C30—C29	120.0 (2)	H32A—C32—H32B	109.470
N21—C31—N29	105.38 (19)	H32A—C32—H32C	109.469
N21—C31—C32	109.3 (2)	H32B—C32—H32C	109.468
N21—C31—C33	110.55 (19)	C31—C33—H33A	109.483
N29—C31—C32	108.00 (18)	C31—C33—H33B	109.470
N29—C31—C33	111.9 (2)	C31—C33—H33C	109.475
C32—C31—C33	111.4 (2)	H33A—C33—H33B	109.464
C1—N1—H1n	114.0 (14)	H33A—C33—H33C	109.468
C11—N1—H1n	114.2 (15)	H33B—C33—H33C	109.468
			
C1—N1—C11—N9	−48.8 (3)	C21—N21—C31—N29	−55.3 (3)
C1—N1—C11—C12	−165.13 (16)	C21—N21—C31—C32	−171.11 (16)
C1—N1—C11—C13	72.6 (3)	C21—N21—C31—C33	65.9 (3)
C11—N1—C1—C2	−156.31 (18)	C31—N21—C21—C22	−150.43 (19)
C11—N1—C1—C10	27.4 (3)	C31—N21—C21—C30	33.5 (3)
C9—N9—C11—N1	51.3 (3)	C29—N29—C31—N21	51.7 (3)
C9—N9—C11—C12	166.88 (18)	C29—N29—C31—C32	168.5 (2)
C9—N9—C11—C13	−70.7 (3)	C29—N29—C31—C33	−68.5 (3)
C11—N9—C9—C8	153.29 (19)	C31—N29—C29—C28	157.1 (2)
C11—N9—C9—C10	−32.0 (3)	C31—N29—C29—C30	−27.6 (3)
N1—C1—C2—C3	−175.13 (17)	N21—C21—C22—C23	−174.14 (19)
N1—C1—C10—C5	174.89 (17)	N21—C21—C30—C25	173.37 (18)
N1—C1—C10—C9	−3.6 (3)	N21—C21—C30—C29	−4.1 (3)
C2—C1—C10—C5	−1.5 (3)	C22—C21—C30—C25	−2.9 (3)
C2—C1—C10—C9	−179.97 (18)	C22—C21—C30—C29	179.64 (19)
C10—C1—C2—C3	1.1 (3)	C30—C21—C22—C23	1.9 (4)
C1—C2—C3—C4	0.3 (4)	C21—C22—C23—C24	−0.3 (4)
C2—C3—C4—C5	−1.2 (4)	C22—C23—C24—C25	−0.3 (4)
C3—C4—C5—C6	−179.83 (19)	C23—C24—C25—C26	178.4 (2)
C3—C4—C5—C10	0.8 (3)	C23—C24—C25—C30	−0.8 (4)
C4—C5—C6—C7	179.12 (19)	C24—C25—C26—C27	−178.6 (2)
C4—C5—C10—C1	0.6 (3)	C24—C25—C30—C21	2.4 (4)
C4—C5—C10—C9	179.05 (18)	C24—C25—C30—C29	179.82 (19)
C6—C5—C10—C1	−178.86 (18)	C26—C25—C30—C21	−176.83 (19)
C6—C5—C10—C9	−0.4 (3)	C26—C25—C30—C29	0.6 (4)
C10—C5—C6—C7	−1.5 (3)	C30—C25—C26—C27	0.6 (4)
C5—C6—C7—C8	1.7 (4)	C25—C26—C27—C28	−1.7 (4)
C6—C7—C8—C9	0.1 (4)	C26—C27—C28—C29	1.6 (4)
C7—C8—C9—N9	172.64 (19)	C27—C28—C29—N29	174.8 (2)
C7—C8—C9—C10	−1.9 (4)	C27—C28—C29—C30	−0.4 (4)
N9—C9—C10—C1	5.7 (3)	N29—C29—C30—C21	1.3 (3)
N9—C9—C10—C5	−172.81 (17)	N29—C29—C30—C25	−176.12 (18)
C8—C9—C10—C1	−179.45 (18)	C28—C29—C30—C21	176.72 (19)
C8—C9—C10—C5	2.1 (3)	C28—C29—C30—C25	−0.7 (4)

Symmetry codes: (i) *x*, −*y*+1/2, *z*−1/2; (ii) −*x*+2, −*y*+1, −*z*+1; (iii) *x*, *y*+1, *z*; (iv) *x*, *y*−1, *z*; (v) −*x*+2, *y*+1/2, −*z*+3/2; (vi) −*x*+2, *y*−1/2, −*z*+3/2; (vii) *x*, −*y*+1/2, *z*+1/2; (viii) −*x*+1, *y*−1/2, −*z*+1/2; (ix) *x*, −*y*+3/2, *z*−1/2; (x) −*x*+1, *y*+1/2, −*z*+1/2; (xi) −*x*+1, −*y*+1, −*z*+1; (xii) *x*, −*y*+3/2, *z*+1/2; (xiii) −*x*+2, −*y*, −*z*+1; (xiv) −*x*+1, −*y*+2, −*z*+1.

### Hydrogen-bond geometry (Å, º)

Cg1, Cg2 and Cg3 are the centroids of the C21–C25/C30, C25–C30 and C5–C10 rings, respectively.

**Table:**

*D*—H···*A*	*D*—H	H···*A*	*D*···*A*	*D*—H···*A*
N21—H21N···N29^v^	0.88 (2)	2.36 (2)	3.229 (4)	170 (2)
N1—H1N···*Cg*1^i^	0.93 (3)	2.93 (3)	3.853 (3)	170 (2)
N9—H9N···*Cg*1	0.88 (4)	2.85 (2)	3.703 (3)	164 (2)
C12—H12*C*···*Cg*2	0.98	2.55	3.521 (3)	172
C26—H26···*Cg*3^iv^	0.95	2.53	3.456 (3)	164

Symmetry codes: (i) *x*, −*y*+1/2, *z*−1/2; (iv) *x*, *y*−1, *z*; (v) −*x*+2, *y*+1/2, −*z*+3/2.
